# Supplementary material for: Phenotypic and Genetic Factors Associated with Absence of Cardiomyopathy Symptoms in PLN:c.40_42delAGA Carriers
Source: J Cardiovasc Transl Res. 2023 Jan 9;16(6):1251–66. doi: 10.1007/s12265-022-10347-5 (PMC10721704; doi:10.1007/s12265-022-10347-5)
Supplement: Supplementary file 3 — Supplementary file3 (DOCX 3.05 MB) [file 12265_2022_10347_MOESM3_ESM.docx]

**Supplementary information**

**Phenotypic and genetic factors associated with absence of cardiomyopathy symptoms in PLN:c.40_42delAGA carriers**

**Supplementary Tables**

S1. Phenotype description

S2. Phenotype associations

S3. Replication of phenotypes

S4. Haplotype analyses

S5. GWAS result

S6. Rare variant analyses

S7. PGS Analysis

S8. Interaction analyses

S9. TWAS

S10. City index and exercises in QRS

**Supplementary Figures**

SF1. Distribution of age in PLN:c.40_42delAGA carriers and non-carriers

SF2. Intensity readings of the genotype calls for the Illumina variant seq-rs397516784 (corresponding to PLN:c.40_42delAGA)

SF3. Clustering of symptomatic and asymptomatic carriers in families

SF4. Unadjusted phenotype distributions according to carrier and symptoms status

SF5. Phenotype distributions according to carrier and symptoms status

SF6. Phenotype distributions for symptomatic and asymptomatic individuals in the ACM/PLN registry

SF7. Differences in number of variants between the haplotypes present within the window of the longest and most frequent haplotype for the 74 PLN:c.40_42delAGA carriers in two screening frames.

SF8. Manhattan and regional association plots for genetic analyses between asymptomatic carriers compared to asymptomatic non-carriers

SF9. Correlations between the variant’s GWAS p-value and its linkage disequilibrium (r^2^) to PLN:c.40_42delAGA for the variants with p-values < 5x10^-4^SF10. Forest plot depicting the effect of PGSs

SF10. Forest plot depicting the effect of PGSs

SF11. Distribution of polygenic scores in the four study groups.

SF12. Distribution of the symptoms in the interaction of PGS_QRS_ with the symptomatic carrier status

SF13. TWAS results in the Heart Left Ventricle

SF14. Correlation between MRI-derived features and ECG features from Verstraelen et al. study and QRS durations in the ACM registry.

SF15. Comparison of the ventricular arrhythmia risk prediction

SF16. Power and sample size calculations for the genetic analyses.

**Supplementary Text**

**Binary analyses of polygenic scores (PGSs)**

As we were concerned that PGSs could have some non-linear effects, we repeated our analyses of PGSs, but focusing on the extremes of the distributions. Specifically, we compared the number of asymptomatic carriers and asymptomatic non-carriers in the upper tail (PGS above the 80^th^ percentile) with those in the rest of the distribution. Likewise, we compared the number of asymptomatic carriers and asymptomatic non-carriers in the lower tail (PGS below the 20^th^ percentile) against the rest. The results for these analyses were consistent with those observed when analyzing the PGS as continuous distribution only for the MRI-lvesv traits (**Supplementary Tables 7D and E**).

**Sensitivity analyses of the significant common variants on chromosome 6 identified by GWAS**

We sought to rule out any potential confounding effects due to the fact that PLN:c.40_42delAGAvariant carriers have a long haplotype that includes common variants that would then be differentially distributed between carriers and non-carriers, regardless of symptoms. The top association on chromosome 6 was observed with SNP rs78559806, which is in low LD (r^2^=0.01, D’=0.83) with the PLN:c.40_42delAGAvariant. When correlating the strength of the associations observed across chromosome 6 (GWAS p-values) with the LD (r^2^) to PLN:c.40_42delAGAvariant, we found a significant correlation (rho=0.92, p<2.2x10^-16^), indicating that the stretch of associations on chromosome 6 is not related to lack or presence of symptoms (**Supplementary Figure 9**).

**Independent effect of QRS and PGS_QRS_ on asymptomatic carriers compared to asymptomatic non-carriers**

We also found a higher predisposition for increased PGS_QRS_ in carriers, which may appear to be in contrast with decreased QRS we observed in asymptomatic carriers. Considering that PGS_QRS_ showed (as expected) a positive correlation with QRS duration (Spearman correlation rho=0.18, p=1.42x10^-182^, using all participants), we hypothesized that the effects of the QRS phenotype and the PGS_QRS_ were independent. To test this hypothesis, we jointly analyzed the PGS_QRS_ and the phenotypic value of QRS duration in one model. Both terms remained significant, and the effect of one was not diminished by the presence of the other in the model (**Supplementary Table 8**), which corroborates that the effects of PGS_QRS_ and QRS on our outcome were truly opposite and independent.

**Associations of QRS duration with participant physical activity measures and urban index of their residence**

We collected residential information for the Lifelines participants and mapped their urban index using information from a previous study.^1^ We then examined the distribution of the urban index for the four groups (asymptomatic carriers, asymptomatic non-carriers, symptomatic carriers, and symptomatic non-carriers) of participants in Lifelines with the following model and OLS:

QRS duration ~ group + urban1+ age + gender + group * urban index

The *group* variable in the model indicates whether a participant is a carrier and whether a participant is symptomatic. The results of the model showed no significant findings for any variable related to the urban index (**Supplementary Table 10A**).

We also collected participant lifestyle information about sports frequency, sedentary duration, exercise duration last week and regular sports duration. The specific parameter names are:

'exercise_pastweek_ach_q_1_1A', 'sports_frequency_ach_q_1_01', 'sports_frequency_ach_q_1_02', 'sports_frequency_ach_q_1_03', 'sports_play_ach_q_1_01', 'sports_play_ach_q_1_02', 'sports_play_ach_q_1_03', 'sports_regular_ach_q_1', 'activity_followup_adu_q_1.B', 'activity_followup_adu_q_1.C', 'exercise_pastweek_ach_q_1_2A', 'sedentary_duration_ach_q_1', 'sport1_hours_ach_q_1', 'sport2_hours_ach_q_1', 'sport3_hours_ach_q_1'.

The explanation for each parameter name can be found in the Lifelines catalog website (See **URL section**). We then assessed the correlation between these exercise variables with the participant's QRS duration values using an OLS model adjusted for gender and age but did not find significant associations between any exercise variables and QRS duration (**Supplementary Table 10B**).

**Ventricular Arrhythmia risk prediction with QRS duration**

We tested the predictability of QRS duration in predicting the VA risk for PLN:c.40_42delAGA carriers in the ACM/PLN registry. For this, we first correlated QRS duration with the predictors used in the model from Verstraelen et al.^2^: the number of negative T waves, presence of low-voltage ECG, PVC count/24 h and LVEF. Based on the correlation results, we identified two predictors that are highly correlated with QRS duration: PVC count/24h and LVEF. We then tested whether QRS duration could partly reflect the predictability for those predictors by comparing the prediction performance between two models: 1) a baseline model that excluded the highly correlated predictors with QRS duration and 2) a model with the predictors used in model (1) and QRS duration. We used leave-one-out AUC value to quantify the prediction performance and determined the significance of the differences between the two models using the DeLong test.^3^ Model construction and evaluation were implemented using Python package scikit-learn v0.22.2.post1^4^ and R package pROC v1.14.0.^3^ The model performance comparison is shown in **Supplementary Figure 15**. Compared to the baseline model, adding QRS duration improve the LOO-AUC from 0.66 to 0.72 (DeLong test p-value=0.007), indicating the strong predictive value of QRS duration.

**Power calculations for the genetic analyses**

We used the rate of unrelated asymptomatic carriers found in our study to perform a power analysis based on a logistic regression model in a range of possible odds ratios (ORs, 1.5-5.0) and a range of different allele frequencies. We then calculated the sample size necessary to achieve 80% power to detect these ORs at MAF = 0.05 in settings with low or high rates of asymptomatic carriers, comparable to those of PLN:c40_42AGA or the mutations in **BRCA1**, respectively (**Supplementary Figure 16**). We used the R package *genpwr*^5^ assuming an additive model and a significance level of 5x10^-8^ for these analyses.

**Supplementary Figures**

**
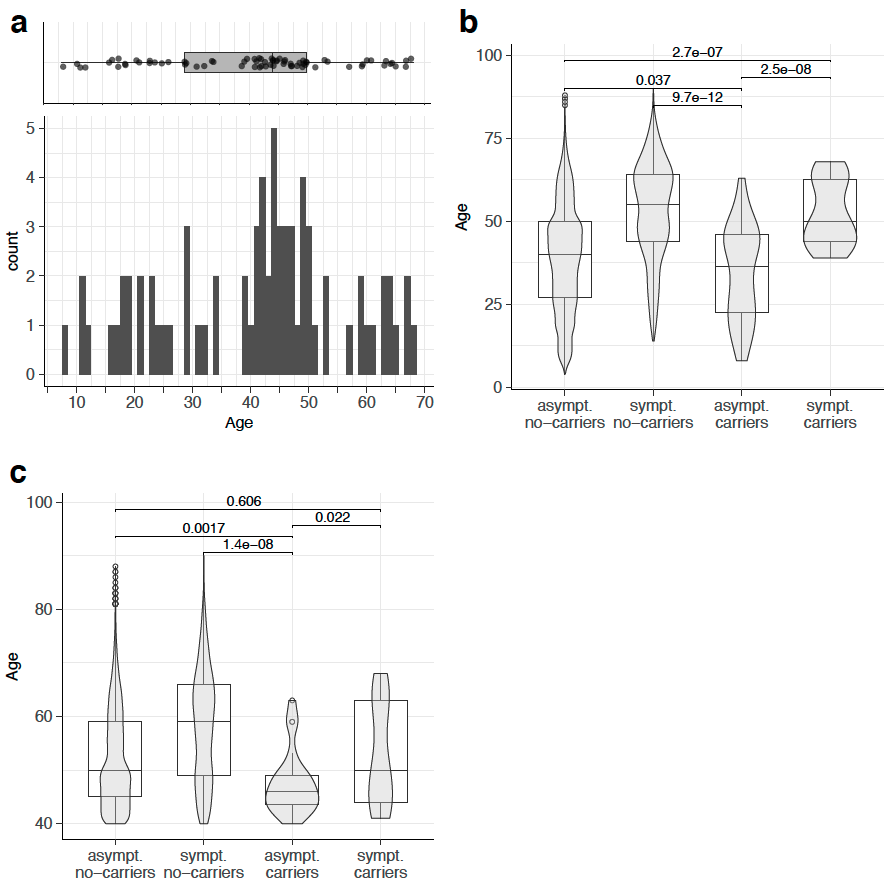
**

**Supplementary Figure 1.** **Distribution of age in PLN:c.40_42delAGA carriers and non-carriers.** a) Boxplot of age distribution (top) and frequency by age (bottom) for the 74 carriers only. b) Boxplot and violin plots for age in each of the four groups: asymptomatic non-carriers (n=34,201), symptomatic non-carriers (n=2,064), asymptomatic carriers (n=48) and symptomatic carriers (n=26). c) Box plots and violin plots for age when including only individuals aged ≥40 years in each of the four groups, asymptomatic non-carriers (n=17,554), symptomatic non-carriers (n=1,705), asymptomatic carriers (n=23) and symptomatic carriers (n=25). *p-values correspond to pairwise t-tests.


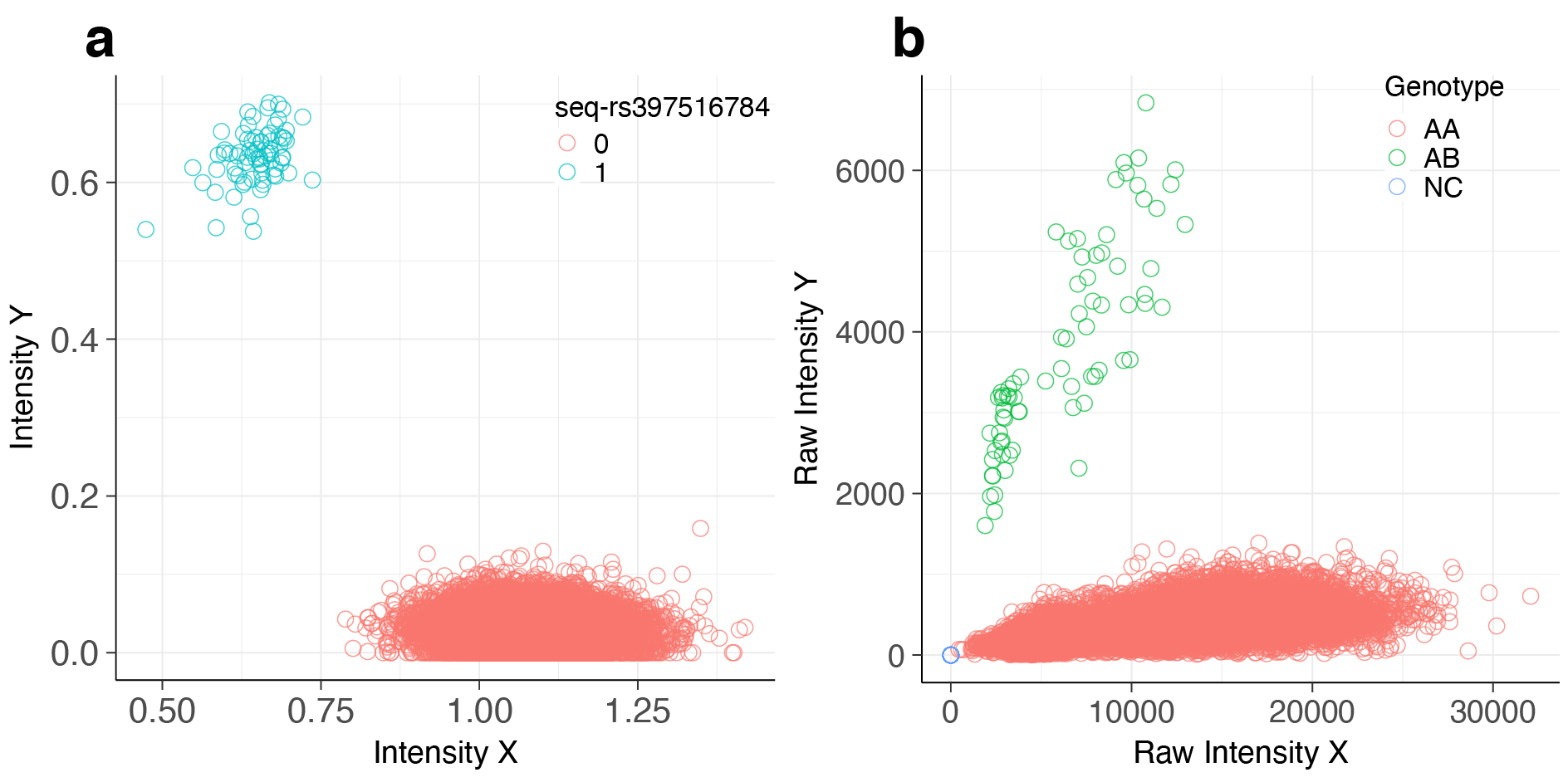


**Supplementary Figure 2.** **Intensity readings of the genotype calls for the Illumina variant seq-rs397516784 (corresponding to PLN:c.40_42AGA).** a) Genotype calls from the Illumina software with normalized intensities for the SNP. The 74 samples with high intensity values would be interpreted as carriers of alternative alleles for this SNP. b) Raw intensity readings show higher Y values for 74 samples, which can be interpreted as the heterozygous genotype (AB). These 74 samples cluster away from those homozygous for the reference allele (AA), which show low intensity values in Y and spread intensities in X. Non-calls (NC) were observed in 14 samples.


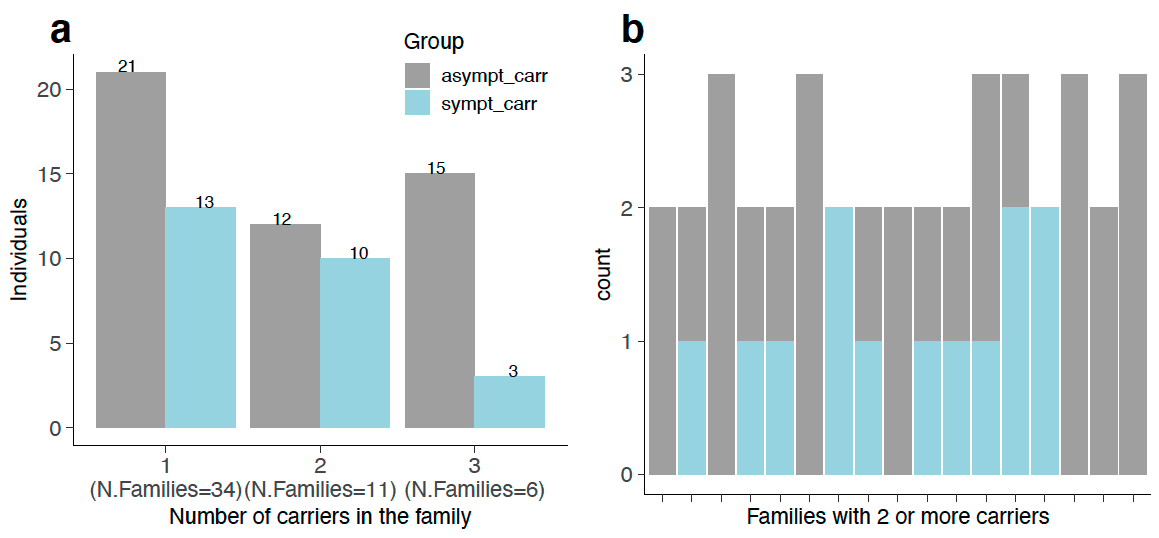


**Supplementary Figure 3. Clustering of symptomatic and asymptomatic carriers in families.** a) Number of symptomatic and asymptomatic individuals per family, with the families represented in the X-axis by the number of carriers they contain. b) Numbers of carriers in the 17 families with more than one carrier. Each frequency bar is one family, and the segments represent the individual carriers, colored according to the presence or absence of symptoms. Only seven symptomatic carriers are in the same family as asymptomatic carriers. asympt_carr. asymptomatic carriers; sympt_carr, symptomatic carriers.


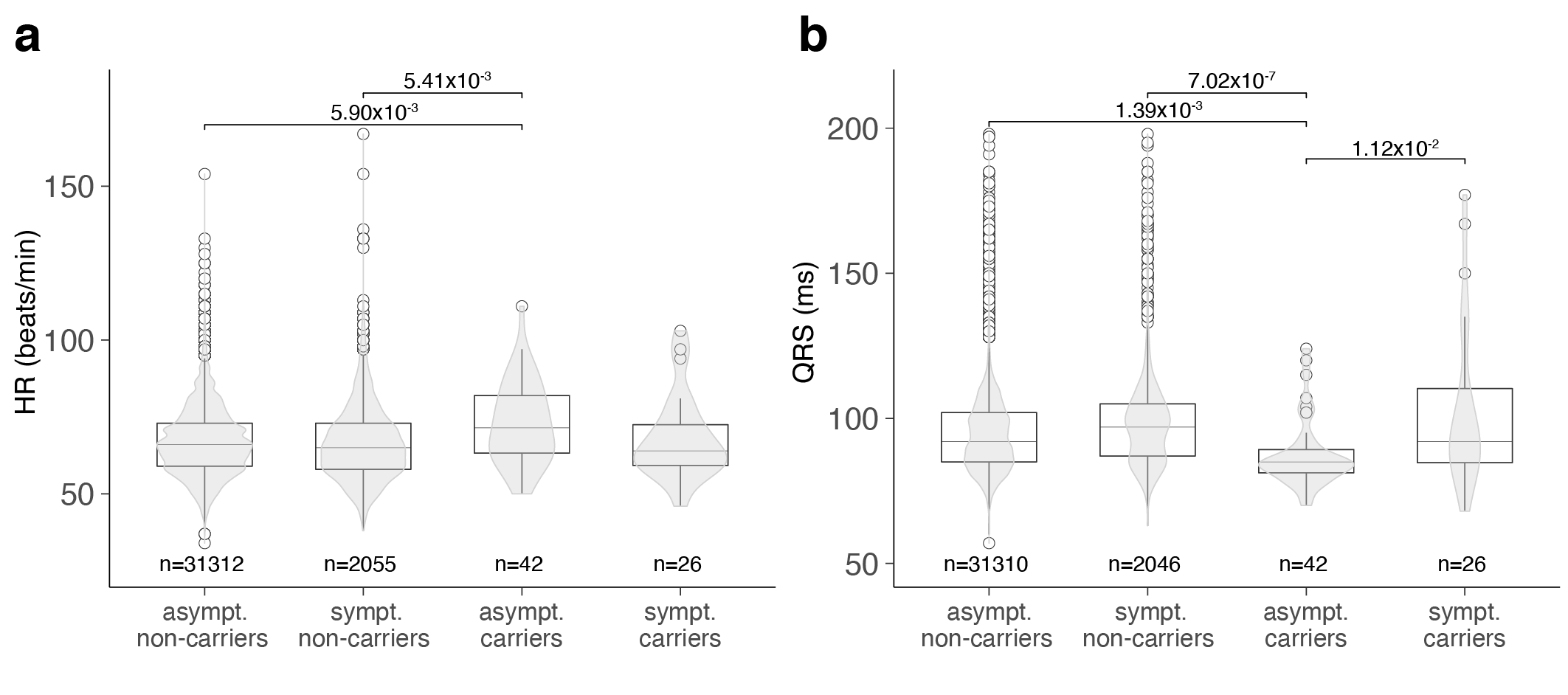


**Supplementary Figure 4. Unadjusted phenotype distributions according to carrier and symptoms status.** Boxplots and violin plots represent quantitative phenotypes as described in **Figure 3.** a) Boxplot and violin plots for unadjusted HR b) Boxplot and violin plots for unadjusted QRS. The p-values here refer to a t-test using the unadjusted traits; only significant p-values are shown.


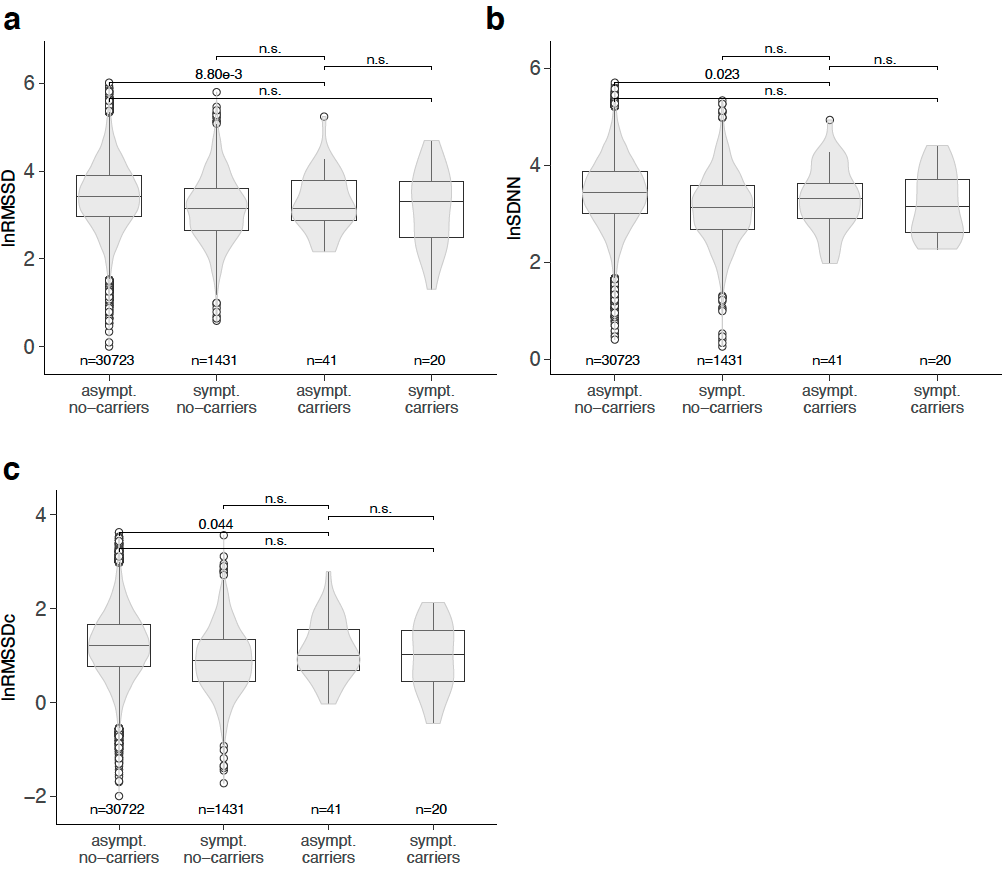


**Supplementary Figure 5. Phenotype distributions according to carrier and symptoms status.** Boxplots and violin plots represent quantitative phenotypes as described in **Figure 3.** a) Boxplot and violin plots for adjusted root mean square of successive differences adjusted by heart rate. b) Boxplot and violin plots for root mean square of successive differences. c) Boxplot and violin plots for the standard deviation of normal-to-normal intervals. The symptomatic carriers group shows decreased values in all three heart rate variability measures that appear to be unique to this group. This difference is apparently not affected by heart rate itself, as shown in (c). p-values correspond to the coefficient of the adjusted trait in the model described in Equation 1.


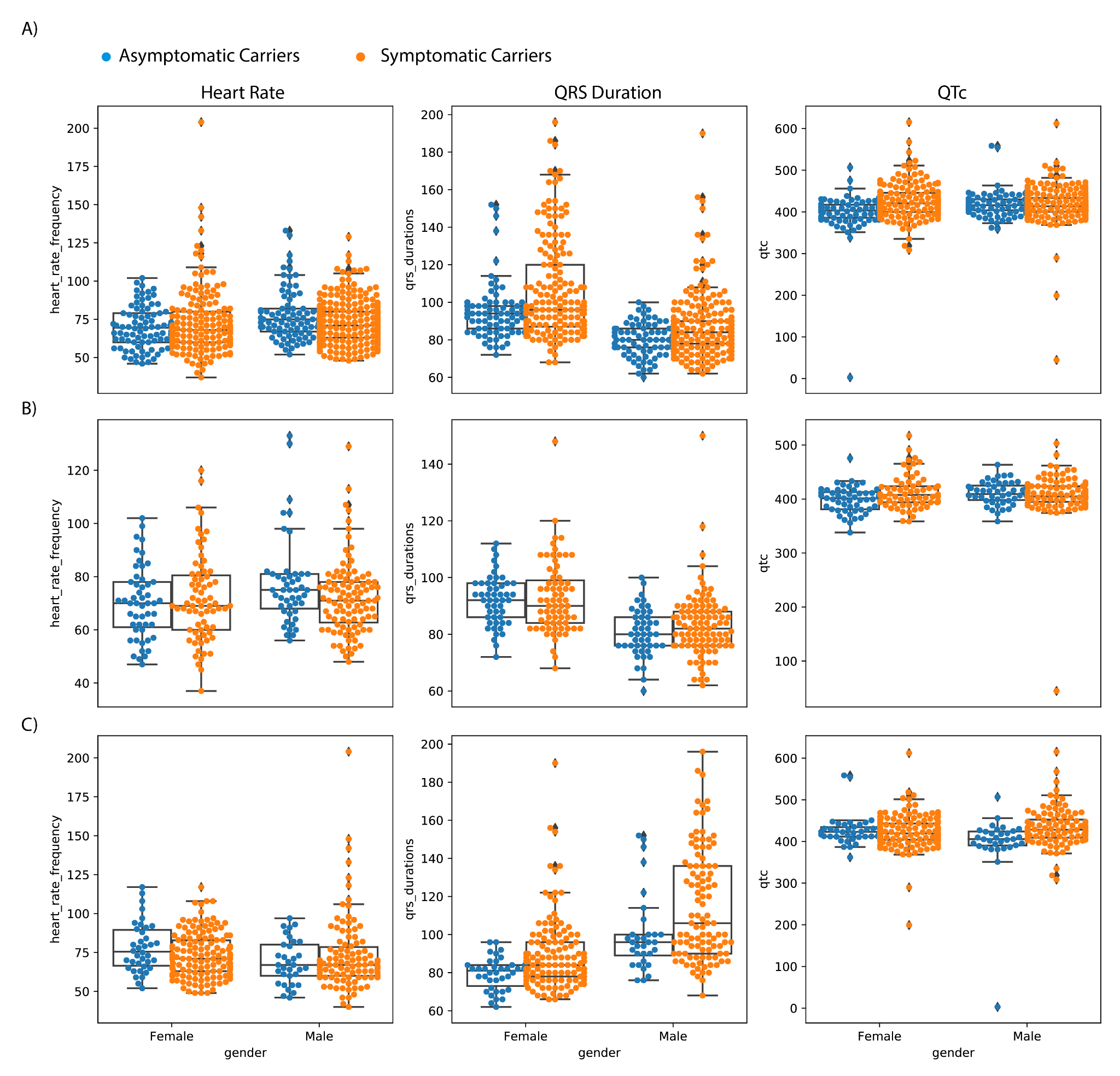


**Supplementary Figure 6.** **Phenotype distributions for symptomatic and asymptomatic individuals in the ACM/PLN registry.** a) Phenotype distribution for the full ACM/PLN registry data. b) Phenotype distribution for younger people (age < 40 years) in the ACM/PLN registry. C) Phenotype distribution for old people (age ≥ 40 years) in the ACM/PLN registry.

**
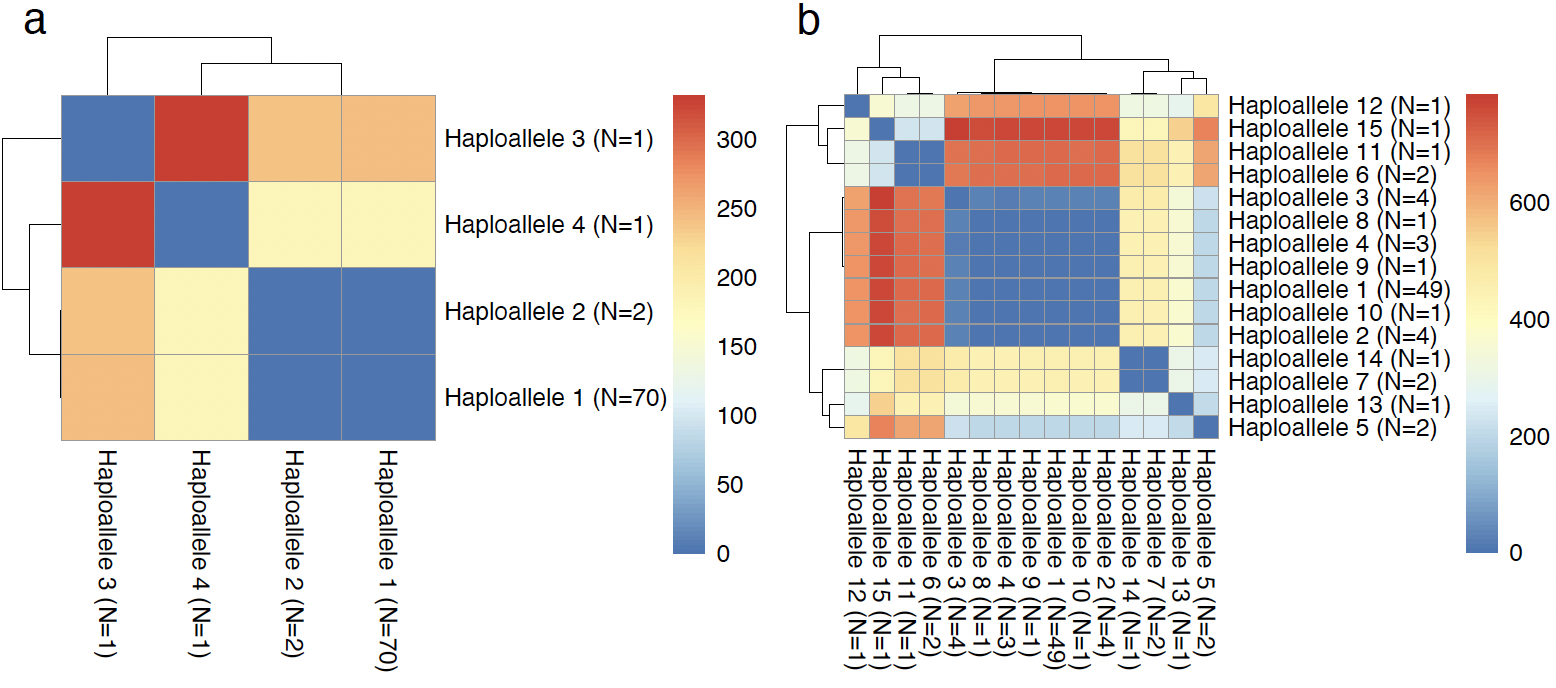
**

**Supplementary Figure 7. Differences in the number of variants between the haplotypes present within the window of the longest most frequent haplotype for the 74 PLN:c.40_42delAGA carriers** **in two screening frames**. a) For a MAF ≥ 0.01 in all the samples. The window comprises 1.38Mb (+605Kb to -770Kb around PLN-p.Arg14del). Haploalelle 2 is closest to the most frequent haploallele (n=70) with 1 SNP of difference, whereas the other haploalleles differ by approximately 330 SNPs. b) For a minor allele count ≥ 4 in the carrier chromosomes. The window comprises 2.25Mb (+245Kb to -1.98Mb), and we did not stop the screening until we reached a split (at least 10 haploallele difference). Therefore, the window extended until the upstream end of the region screened. Other haploalleles differ from the longest most frequent haploallele (n=49) in a range of 2 and 780 SNPs.


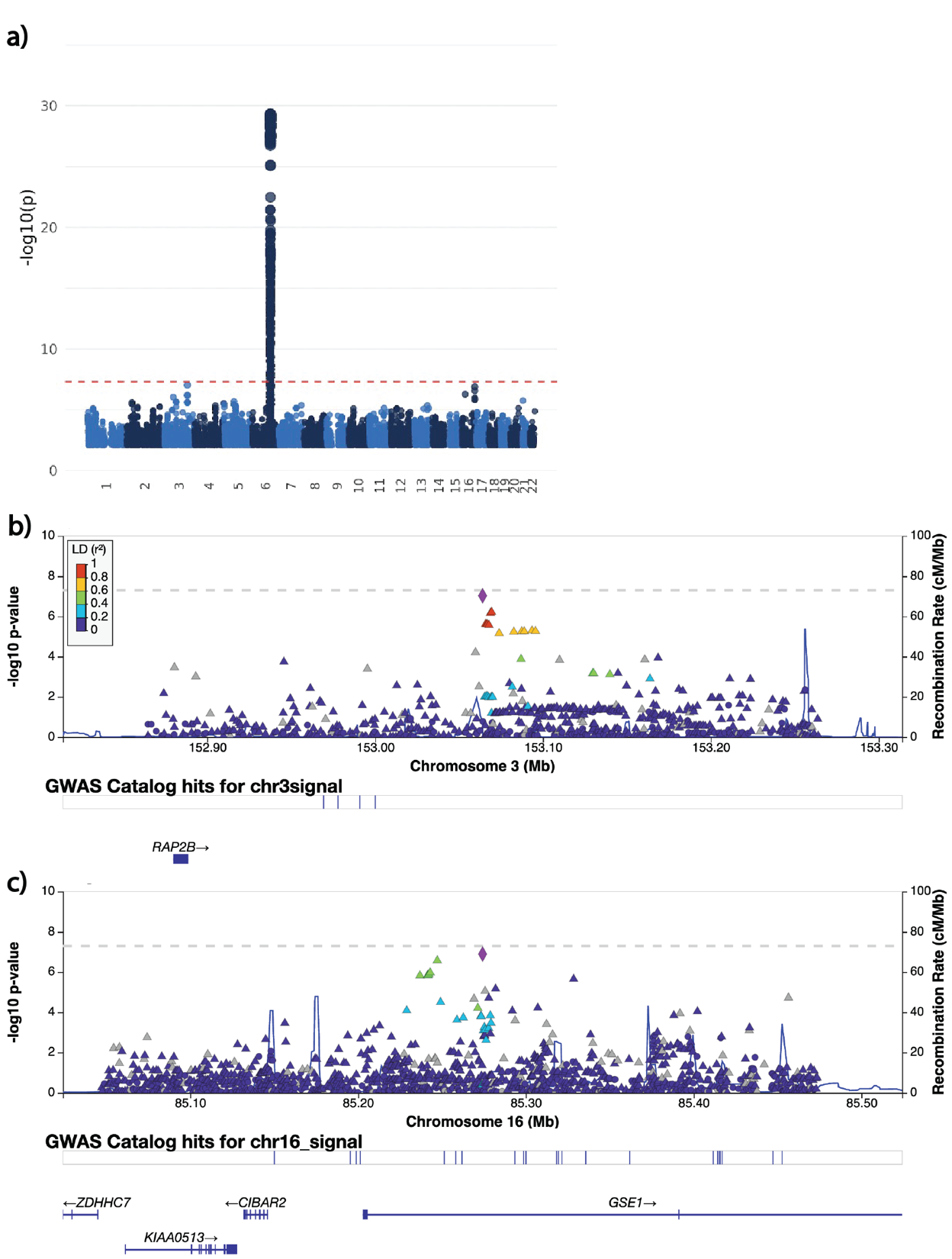


**Supplementary Figure 8. Manhattan and regional association plots for genetic analyses between asymptomatic carriers compared to asymptomatic non-carriers.** a) Genome-wide comparison. Dotted red line corresponds to the genome-wide significance threshold (p=5x10^-8^). b) Locus-zoom plot for 200Kb around rs6768326 in chromosome 3. c) Locus-zoom plot for 200Kb around rs112525682 in chromosome 16. Colors indicate LD according to the legend in c). LD measures correspond to EUR population.


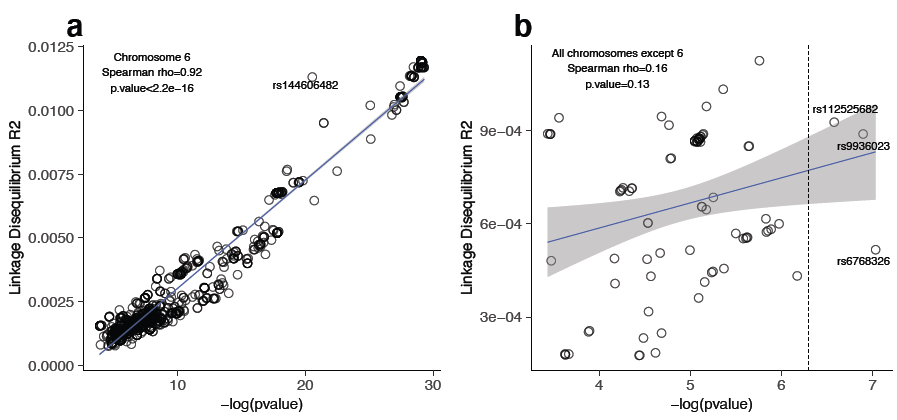


**Supplementary Figure 9. Correlations between the variant’s GWAS p-value and its linkage disequilibrium (r^2^) to PLN:c.40_42delAGA for variants with p-values < 5x10^-4^.** Each point represents a genetic variant. a) Correlation for SNPs located in chromosome 6 only. b) Correlation with SNPs located in the rest of the genome. SNPs outside of this correlation and with a p-value < 5x10^-7^ (dashed line) were labeled and further analyzed. Regression lines are depicted in blue.


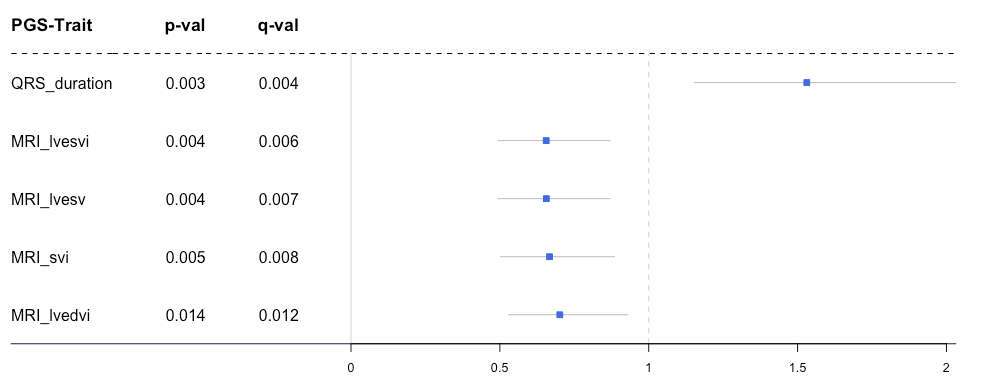


**Supplementary Figure 10**. **Forest plot depicting the effect of PGSs.** Squares represent the effect size (beta). Lines represent standard error. p-val, p-value for the independent association; q-val, false discovery rate for conjoined analysis of all the traits, with 1000 permutations for each of the 97 PGS-traits.


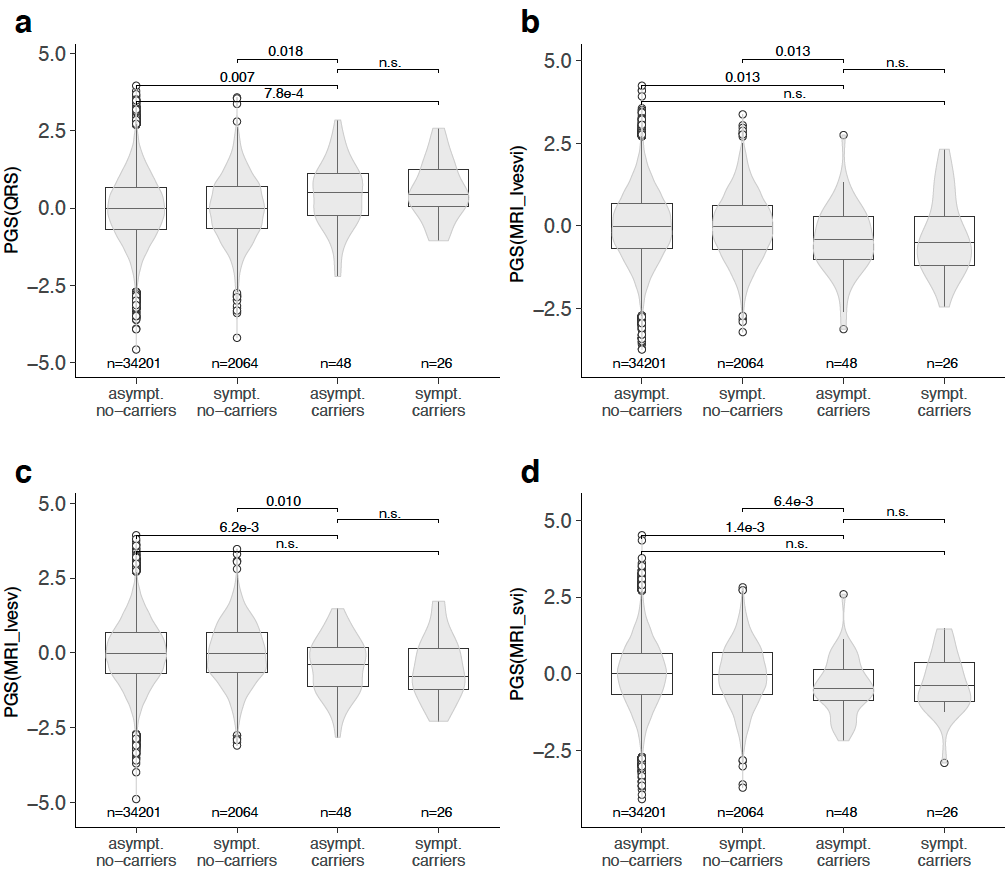


**Supplementary Figure 11.** **Distribution of polygenic scores in the four study groups.** Violin and boxplots represent summary statistics of each PGS as described in **Figure 3**. Comparisons are shown for a) PGS_QRS_, b) PGS_lvesvi_, c) PGS_lvesv_ and d) PGS_svi_. The distribution of asymptomatic carriers is not different from that of the symptomatic carriers, while the difference compared to the symptomatic non-carriers for both groups of carriers has the same direction. P-values correspond to the regression coefficient of the adjusted trait in the model described in equation 2, comparing the groups indicated by the ticks. asympt, asymptomatic; sympt, symptomatic.


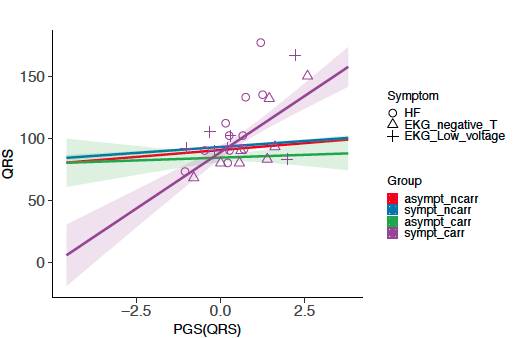


**Supplementary Figure 12. Distribution of symptoms in the interaction of PGS_QRS_ with the symptomatic carrier status.** Regression lines represent the partial correlation between PGS_QRS_ and QRS for each of the groups. Symbols represent the symptoms only for symptomatic carriers, ordered according to severity as in the legend from highest to lowest. For individuals with more than one symptom, only the most severe is depicted. There is no apparent pattern in the severity in this interaction. HF, heart failure, EKG_negative_T, negative T in the electrocardiogram; EKG_Low_voltage, low voltages or microvoltages in the electrocardiogram.


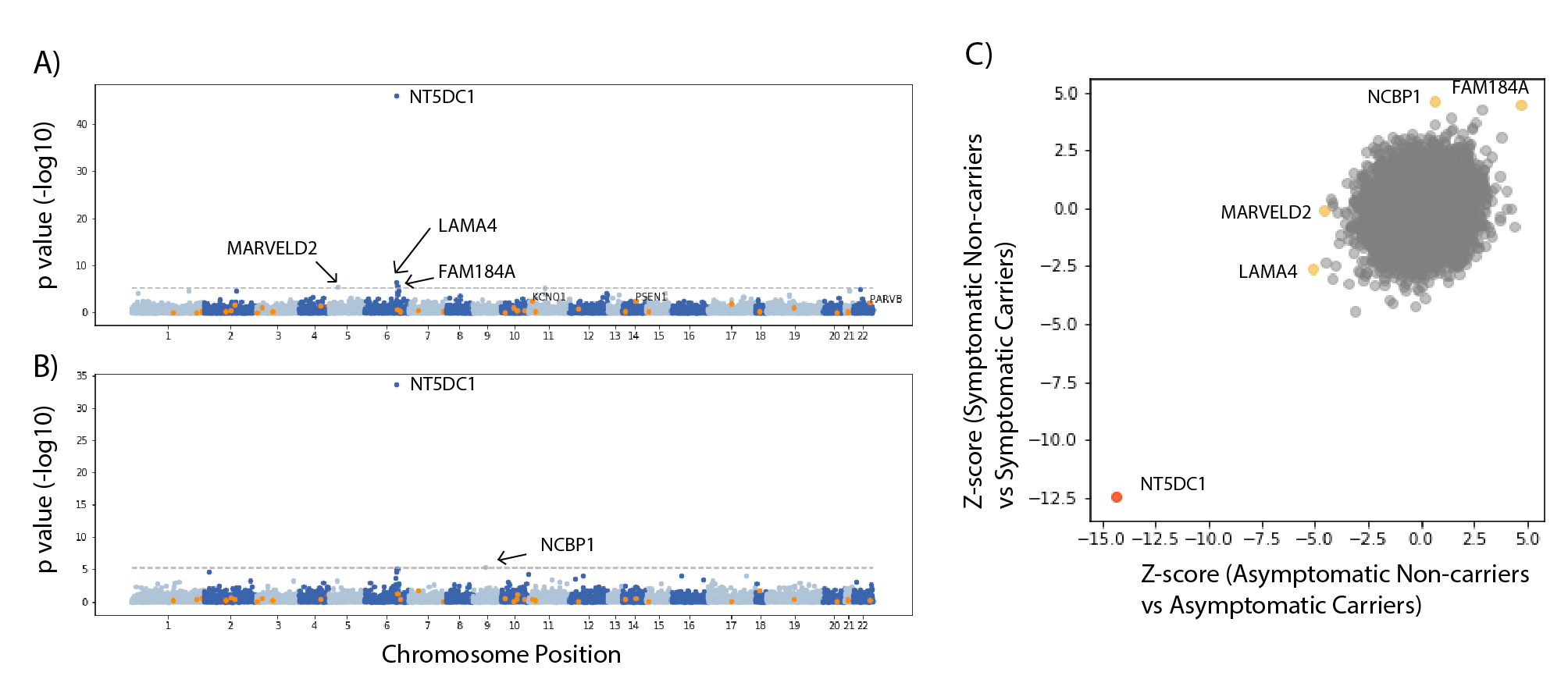


**Supplementary Figure 13**. **TWAS results in Heart Left Ventricle.** a) For asymptomatic carriers compared to asymptomatic non-carriers. b) For symptomatic carriers compared to symptomatic non-carriers. Significance level is determined as -log(p) < 5.18. Each dot in the figure represents a gene. Orange dots are genes in the cardiomyopathy panel. c**)** Comparison between the z-scores of TWAS results for asymptomatic carriers and those for symptomatic carriers. Dark orange dots indicate genes significant in both TWAS studies. Light orange dots indicate genes significant in only one TWAS study.


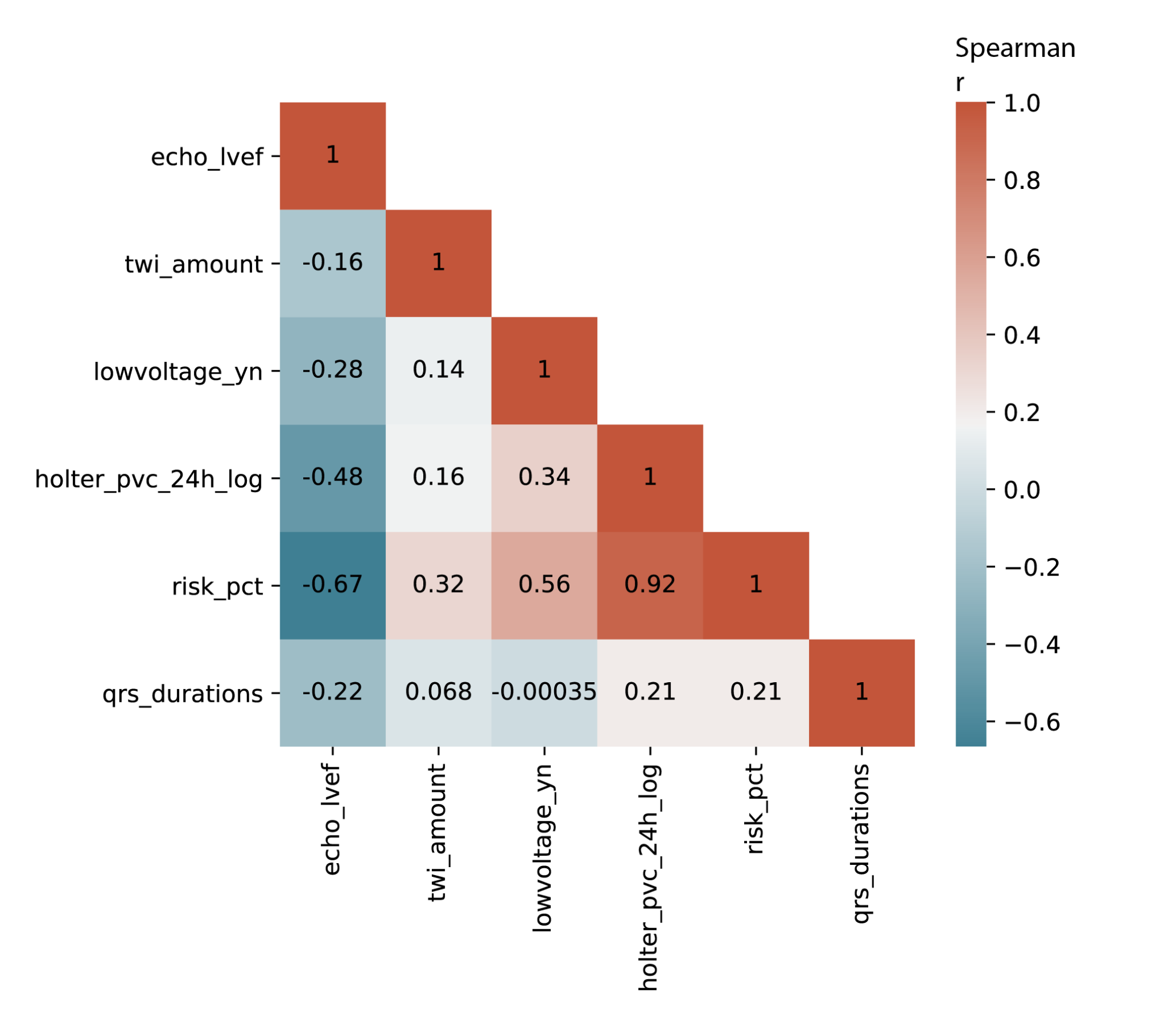


**Supplementary Figure 14.** **Correlation between MRI-derived features and ECG features from the Verstraelen et al. study and QRS durations in the ACM/PLN registry.** Box color indicates Spearman r value.


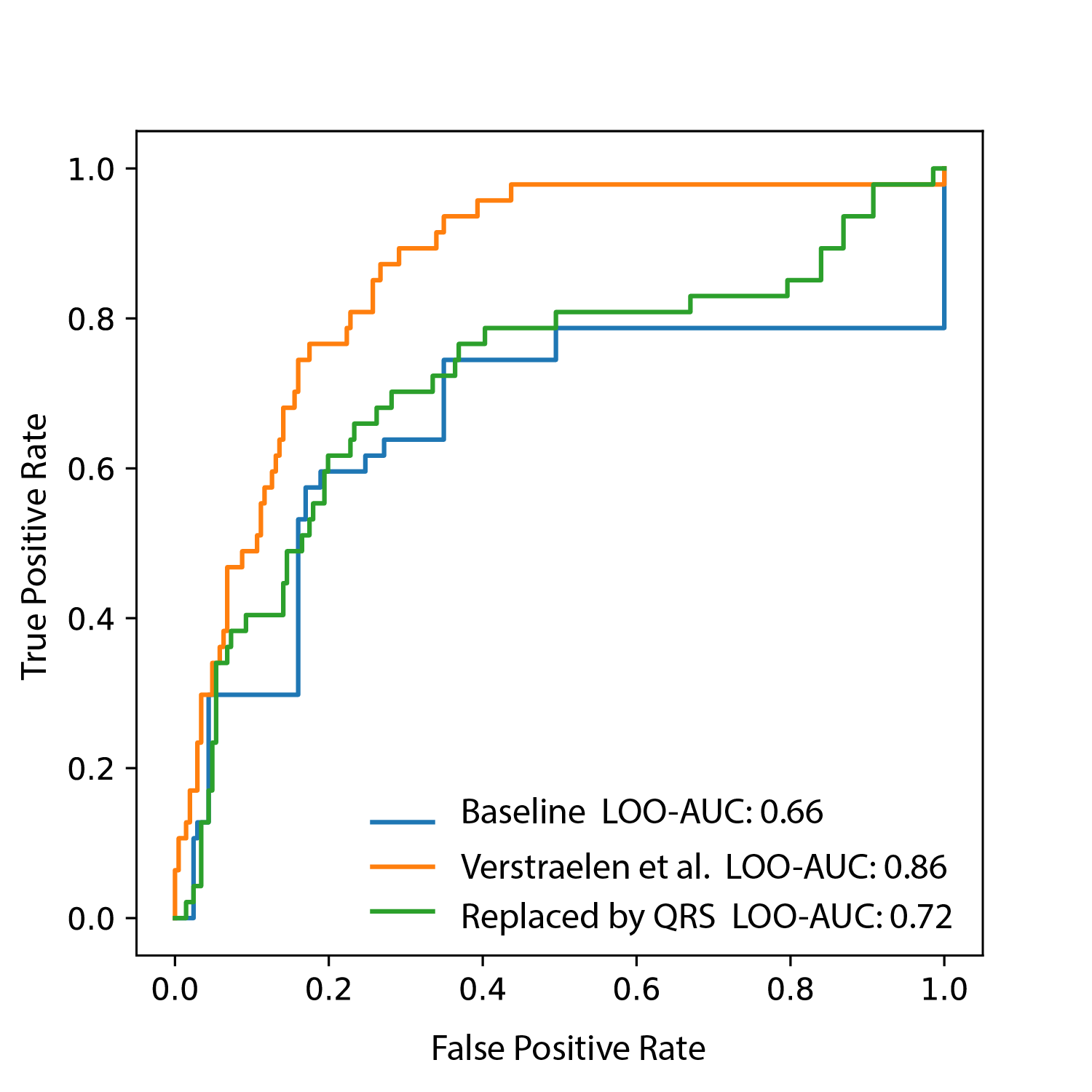


**Supplementary Figure 15**. **Comparison of the ventricular arrhythmia risk prediction.** The ROC curve for Verstaelen et al., study refers to the model that was trained with the full set of features, including: LVEF, 24-h PVC counts, T wave inversion and the presence of low voltage ECG. “Replaced by QRS” indicates that the model is obtained using QRS duration, T wave inversion and the presence of low voltage ECG. “Baseline” indicates that this model is obtained using only T wave inversion and the presence of low voltage ECG. The model performance is indicated by the leave-one-out (LOO) AUC values.


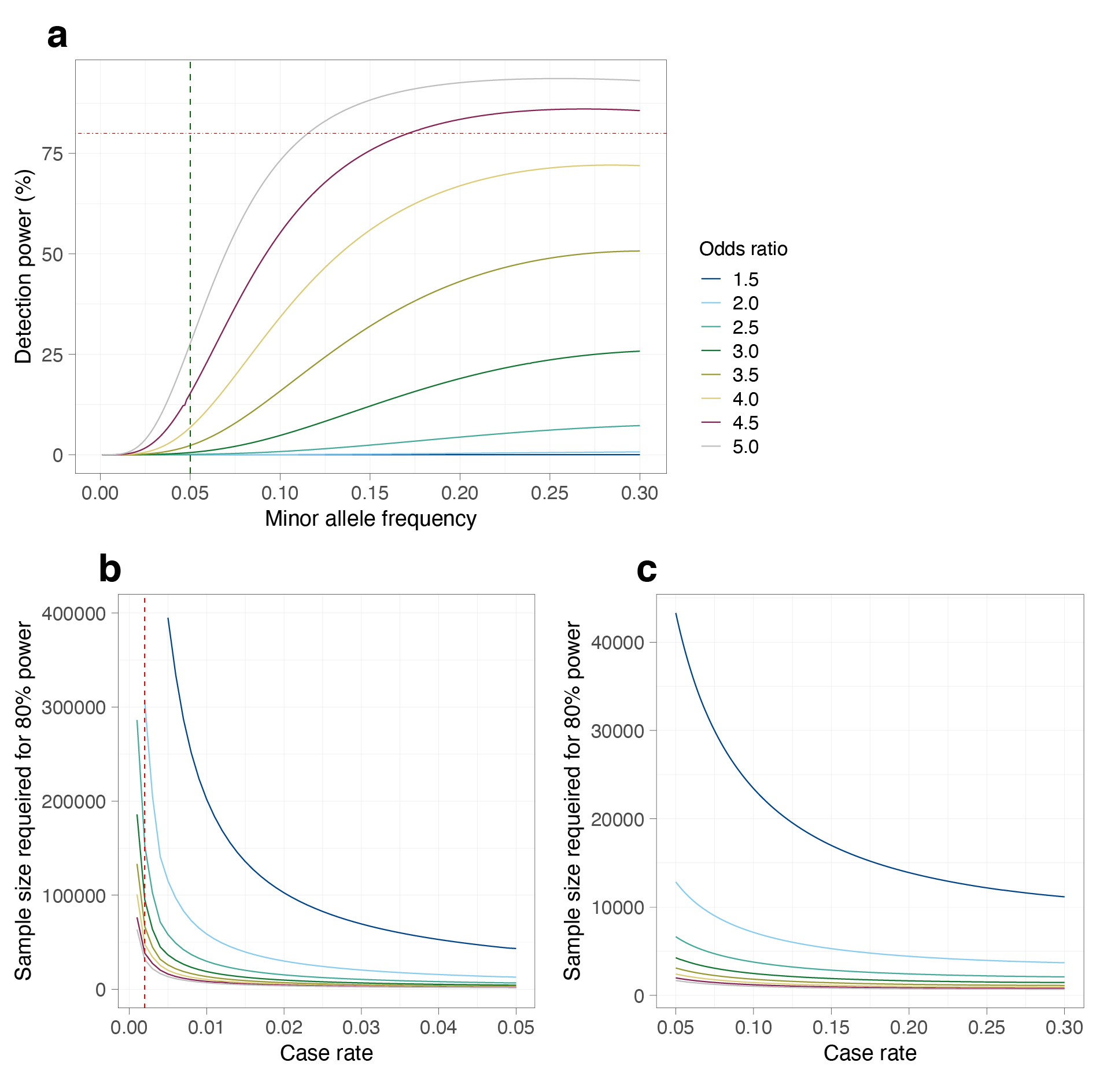


**Supplementary Figure 16. Power and sample size calculations for the genetic analyses.** The estimations were performed **for** line indicates the 80% power threshold, and the green vertical line indicates the 0.05 MAF threshold used for the selection of variants in the GWAS. **b)** Sample sizes required to detect a specific OR with 80% power for variants of MAF = 0.05 in rates of asymptomatic carriers between 0.001 and 0.05. The red vertical line indicates the rate observed in this study. Specific OR are depicted with lines colored according to the color-coding legend in panel a). **c)** Same as b) but for rates of asymptomatic carriers between 0.05 and 0.30 to represent more common monogenic diseases.

**References:**

1. Gacesa, R. *et al.* Environmental factors shaping the gut microbiome in a Dutch population. *Nature* **604**, 732–739 (2022).

2. Verstraelen, T. E. *et al.* Prediction of ventricular arrhythmia in phospholamban p.Arg14del mutation carriers–reaching the frontiers of individual risk prediction. *European Heart Journal* **42**, 2842–2850 (2021).

3. Robin, X. *et al.* pROC: an open-source package for R and S+ to analyze and compare ROC curves. *BMC Bioinformatics* **12**, 77 (2011).

4. Pedregosa, F. *et al.* Scikit-learn: Machine Learning in Python. *Journal of Machine Learning Research* **12**, 2825–2830 (2011).

5. Moore, C. M., Jacobson, S. A. & Fingerlin, T. E. Power and Sample Size Calculations for Genetic Association Studies in the Presence of Genetic Model Misspecification. *Hum Hered* **84**, 256–271 (2019).
